# Diabetes duration modifies the association between AIP and MAFLD in type 2 diabetes

**DOI:** 10.3389/fendo.2026.1952511

**Published:** 2026-10-06

**Authors:** Jiayu Ma, Xiaokang Hu, Xiaohe Liang, Kangtao Jin

**Affiliations:** 1 Department of Endocrinology, Affiliated Dongyang Hospital of Wenzhou Medical University, Dongyang, China; 2 Vascular Signalling, Molecular Cardiology, Department of Cardiology I-Coronary and Peripheral Vascular Disease, Heart Failure, University Hospital Münster, Münster, Germany

**Keywords:** atherogenic index of plasma, diabetes duration, insulin resistance, metabolic dysfunction-associated fatty liver disease, risk prediction, type 2 diabetes mellitus

## Abstract

**Background:**

Metabolic dysfunction-associated fatty liver disease (MAFLD) is highly prevalent in patients with type 2 diabetes mellitus (T2DM). Although several insulin resistance (IR)-related indices have been proposed as surrogate markers, their comparative performance and the influence of diabetes duration remain unclear. We compared the associations of eight IR-related indices with MAFLD and evaluated the modifying effect of diabetes duration.

**Methods:**

This retrospective cross-sectional study included 5,996 patients with T2DM, of whom 3,087 had MAFLD. Logistic regression was used to evaluate the associations of eight IR-related indices (TyG, TyG-BMI, METS-IR, CHG, CTI, HOMA-IR, SHR, and AIP) with prevalent MAFLD. To facilitate comparison across indices measured on different scales, odds ratios (ORs) were additionally estimated per 1-standard deviation (SD) increase. Receiver operating characteristic (ROC) curve analysis was used to assess discriminative performance. LASSO regression was used for covariate selection, followed by multivariable logistic regression, restricted cubic spline (RCS) analysis, and interaction analysis. Internal validation of the multivariable model was performed using 1,000 bootstrap resamples.

**Results:**

All eight IR-related indices were significantly associated with prevalent MAFLD. After standardization, AIP showed the largest association estimate per 1-SD increase (OR = 2.137, 95% CI: 2.007–2.275). AIP also showed the highest, although moderate, discriminative performance among the eight indices (AUC = 0.692). After LASSO-based covariate selection and multivariable adjustment, AIP remained independently associated with prevalent MAFLD (OR = 4.968, 95% CI: 4.100–6.035). Adjusted RCS analysis indicated a nonlinear association between AIP and prevalent MAFLD (P for overall < 0.001; P for nonlinearity = 0.040). The revised multivariable model had an apparent AUC of 0.758 (95% CI: 0.746–0.771), with an optimism-corrected AUC of 0.756 after bootstrap internal validation. Significant interactions were observed between AIP and diabetes duration when duration was modeled continuously (P for interaction = 0.031) and categorically (<5 vs. ≥5 years; P for interaction = 0.001).

**Conclusions:**

Among the eight IR-related indices evaluated, AIP showed the largest standardized association with prevalent MAFLD and the highest discriminative performance in patients with T2DM. The association between AIP and MAFLD may vary according to diabetes duration.

## 1 Background

Metabolic dysfunction-associated fatty liver disease (MAFLD) is highly prevalent among patients with type 2 diabetes mellitus (T2DM) and represents one of the most common metabolic complications in this population (1). Previous studies have shown that fatty liver disease affects approximately 70% of patients with T2DM and is closely associated with advanced liver fibrosis, cardiovascular disease, chronic kidney disease, and increased all-cause and cardiovascular mortality (2, 3). Therefore, early identification of MAFLD in patients with T2DM is clinically important for risk stratification and timely intervention (4).

Insulin resistance is a central mechanism linking T2DM and MAFLD (5). It promotes hepatic lipid accumulation through increased adipose tissue lipolysis, excessive free fatty acid delivery to the liver, enhanced hepatic *de novo* lipogenesis, and impaired lipid oxidation. Accordingly, several insulin resistance-related surrogate indices, including the triglyceride-glucose index (TyG) (6), the cholesterol, high density lipoprotein, and glucose (CHG) index (7), C-reactive protein-triglyceride-glucose index (CTI) (8), and atherogenic index of plasma (AIP) (9), have been proposed as convenient tools for identifying metabolic dysfunction and fatty liver disease.

AIP, calculated as log10(TG/HDL-C), is a lipid-based marker reflecting atherogenic dyslipidemia, insulin resistance, and cardiovascular risk. Given the close relationship among hepatic steatosis, lipid metabolic disorders, insulin resistance, and atherosclerotic risk, AIP may serve as a clinically useful marker for identifying MAFLD (10). Several studies have reported significant associations between AIP and NAFLD/MAFLD, including studies conducted in patients with T2DM (11). Moreover, direct comparisons between AIP and other insulin resistance-related indices remain limited, and whether diabetes duration modifies the association between AIP and MAFLD has not been fully clarified.

Therefore, in this large retrospective study of hospitalized patients with T2DM, we aimed to systematically compare the associations and discriminative performance of multiple insulin resistance-related indices, including TyG, CHG, CTI, and AIP, for prevalent MAFLD. We further investigated the dose–response relationship between AIP and MAFLD, evaluated the modifying effect of diabetes duration, and developed a multivariable model integrating AIP with relevant clinical characteristics to improve discrimination of prevalent MAFLD.

## 2 Materials and methods

### 2.1 Study population

This retrospective cross-sectional study consecutively enrolled patients with diabetes mellitus who were hospitalized in the Department of Endocrinology at Affiliated Dongyang Hospital of Wenzhou Medical University between January 2016 and December 2025. A total of 9,416 patients were initially screened.

Eligible participants met the following inclusion criteria: (1) diagnosis of T2DM according to the Chinese Guidelines for the Prevention and Treatment of Type 2 Diabetes Mellitus (12); (2) age ≥18 years; (3) availability of liver ultrasonography required for the diagnosis of MAFLD during hospitalization; (4) complete laboratory measurements, including fasting blood glucose and lipid profiles, allowing calculation of insulin resistance-related indices, including the AIP, TyG, CHG, CTI; and (5) complete clinical data.

The term MAFLD was retained because the study population and outcome were defined according to the MAFLD diagnostic framework. As detailed quantitative data on alcohol consumption were unavailable in this retrospective study, participants could not be reliably reclassified according to the newer SLD nomenclature (13).

Patients were excluded if they had (1) type 1 diabetes mellitus, gestational diabetes mellitus, or other specific types of diabetes; (2) viral hepatitis, autoimmune liver disease, alcoholic liver disease, or other chronic liver diseases; (3) malignant tumors; (4) severe hepatic or renal dysfunction; or (5) missing key clinical, laboratory, or imaging data required for the present study.

Among the 9,416 initially screened patients, 6 were excluded because of type 1 diabetes mellitus, gestational diabetes mellitus, or other specific types of diabetes; 185 were excluded because of viral hepatitis, autoimmune liver disease, alcoholic liver disease, or other chronic liver diseases; 475 were excluded because of malignant tumors or severe hepatic and renal dysfunction; and 2,754 were excluded because of missing key clinical, laboratory, or imaging data that precluded calculation of the primary study variables. Ultimately, 5,996 patients with T2DM were included in the final analysis. The study flowchart is presented in **Figure 1**.

**Figure 1:**
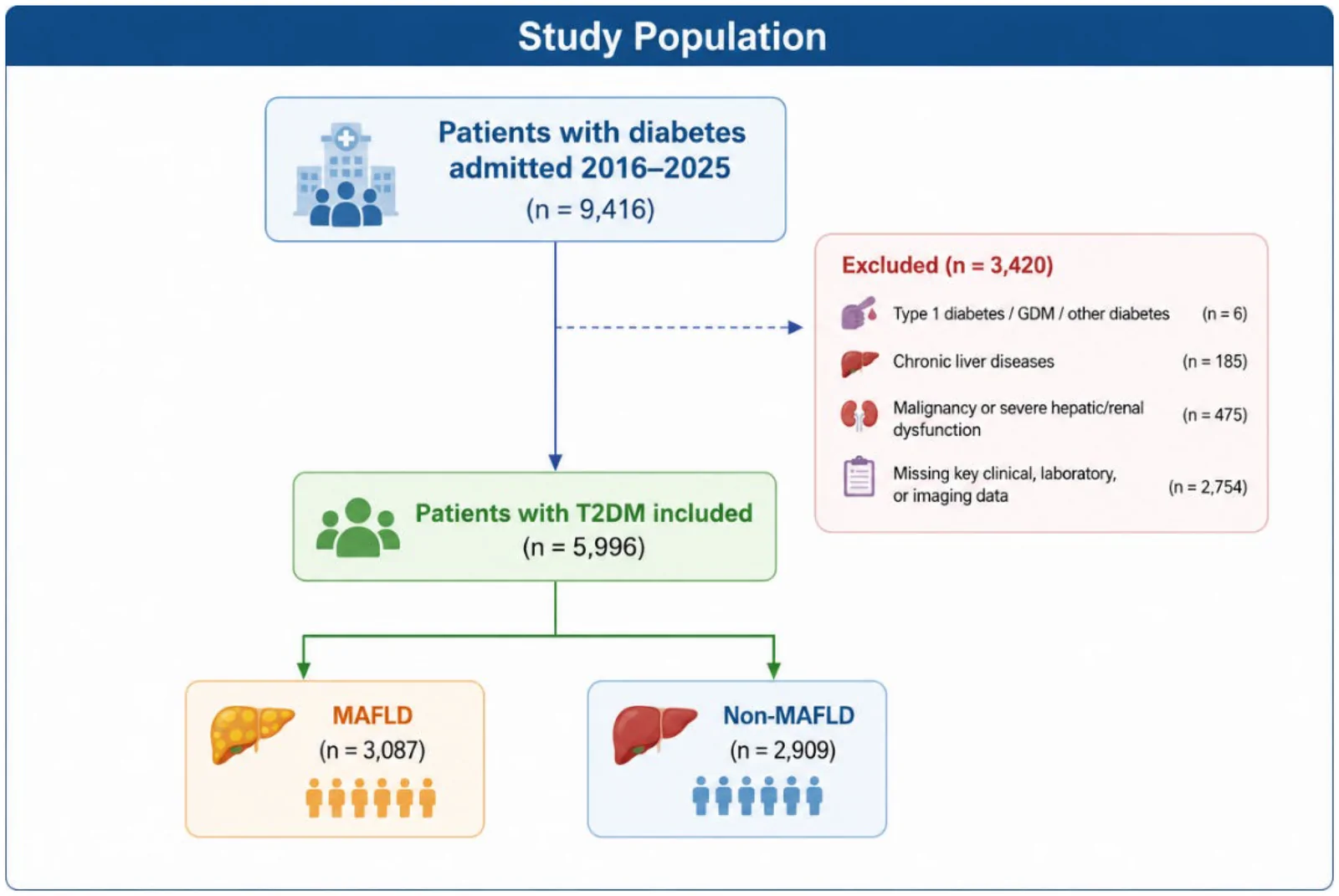
Flow diagram of patient selection.

### 2.3 Data collection

Clinical data were retrospectively extracted from the hospital electronic medical record database. The collected variables included demographic characteristics, anthropometric measurements, laboratory parameters, liver ultrasonography findings, diabetes-related complications, and medication use.

Demographic characteristics included age, sex, diabetes duration, family history of diabetes, smoking status, and alcohol consumption. Anthropometric measurements included height, weight, BMI, SBP, and DBP. Laboratory measurements included glucose metabolism, lipid profile, liver function, renal function, complete blood count, inflammatory markers, tumor markers, and other biochemical parameters. Information regarding diabetic complications and medication use was also collected. Detailed laboratory variables included FBG, FINS, HbA1c, TG, TC, HDL-C, LDL-C, Ca, P, UA, TP, IBIL, ALT, AST, GGT, Scr, eGFR, WBC, NEUT, LYM, RBC, HGB, PLT, hs-CRP, and CEA. Diabetes duration was defined as the interval between the self-reported date of T2DM diagnosis and the date of hospitalization. The information was obtained during routine physician interviews conducted by endocrinologists and documented in the electronic medical records.

Based on these clinical and laboratory parameters, insulin resistance-related surrogate indices were subsequently calculated as described below.

### 2.4 Calculation of insulin resistance-related surrogate indices

Insulin resistance-related surrogate indices were calculated using previously validated equations. Plasma glucose and lipid concentrations were converted from mmol/L to mg/dL when required using standard conversion factors (glucose ×18, triglycerides ×88.57, total cholesterol ×38.67, and high-density lipoprotein cholesterol ×38.67).

The indices were calculated as follows:

-
TYG=ln[TG(mg/dL)×FBG(mg/dL)/2] (14);
-
$\text{TYG}-\text{BMI}=TyG\times BMI\left(kg/m^{2}\right)$ (15);
-
$\text{METS}-\text{IR}=ln[\left(2\times FBG(mg/dL))+TG(mg/dL\right]\times BMI(kg/m^{2})/ln[HDL-C(mg/dL)]$ (16);
-
CHG=ln[(TC(mg/dL)×FBG(mg/dL))/(2×HDL−C(mg/dL))] (17);
-
CTI=0.412×ln(hs−CRP)+ln[(TG(mg/dL)×FBG(mg/dL))/2] (18);
-
HOMA−IR=FBG(mmol/L)×fasting insulin(μU/mL)/22.5 (19);
-
SHR=FBG(mg/dL)/[28.7×HbA1c(%)−46.7] (20);
-
$\text{AIP}=\log_{10}[TG(mmol/L)/HDL-C(mmol/L)]$ (21).

The components and principal metabolic characteristics of the eight indices are summarized in **Supplementary Table S1**.

### 2.5 Statistical analysis

All statistical analysis were performed using R software (version 4.5.3) and Python (version 3.10.6). Continuous variables were expressed as mean ± standard deviation (SD) or median (interquartile range, IQR), as appropriate, and compared using the independent-samples t test or Mann–Whitney U test. Categorical variables were presented as frequencies (percentages) and compared using the chi-square test or Fisher’s exact test.

Baseline characteristics and eight insulin resistance-related indices were compared between patients with and without MAFLD. Univariable logistic regression was used to evaluate the associations between each index and prevalent MAFLD. Because the indices were measured on different scales, standardized ORs (per 1-SD increase) were additionally calculated to facilitate comparison across indices. ROC curves were used to assess discriminative performance, and AUCs were compared using the DeLong test.

LASSO regression was used for covariate selection, with variables with non-zero coefficients retained for subsequent multivariable logistic regression (**Supplementary Table S2**). AIP was considered the primary exposure and was not subjected to LASSO selection. The association between AIP and prevalent MAFLD was further examined using adjusted restricted cubic spline (RCS) analysis with four knots. Model discrimination, calibration, and potential clinical utility were evaluated using ROC curves, calibration curves, decision curve analysis (DCA), and clinical impact curves (CIC). Internal validation was performed using 1,000 bootstrap resamples.

Interaction analysis were performed to examine whether diabetes duration modified the association between AIP and prevalent MAFLD, with diabetes duration modeled both continuously and categorically (<5 vs. ≥5 years). Sensitivity analysis were additionally performed using three duration categories (<5, 5–10, and ≥10 years). The AUCs of AIP between the <5-year and ≥5-year groups were compared using the DeLong test.

At the initial screening stage, patients lacking data essential for calculating the eight indices or assessing fatty liver were excluded from the primary analysis. Variable-level missingness before exclusion was quantified, and available baseline characteristics were compared between included and excluded participants using standardized mean differences (SMDs) (**Supplementary Table S3**). Among patients eligible for the primary analysis, residual missingness in other covariates was <3%; therefore, complete-case analysis was performed without imputation. All tests were two-sided, and P <0.05 was considered statistically significant.

### 2.6 Ethics approval and consent to participate

This study was approved by the Ethics Committee of Affiliated Dongyang Hospital of Wenzhou Medical University (Approval No. 2026-YX-168). Given the retrospective nature of the study, the requirement for informed consent was waived by the Ethics Committee. All procedures involving human participants were conducted in accordance with the ethical standards of the institutional research committee and the Declaration of Helsinki and its later amendments.

## 3 Results

### 3.1 Baseline characteristics according to MAFLD status

A total of 5,996 patients with T2DM were included in the analysis, of whom 3,087 (51.5%) had MAFLD and 2,909 (48.5%) did not (**Table 1**).

**Table 1:** Comparison of baseline characteristics between patients with and without MAFLD.

Characteristics		Total (n=5996)	No-MAFLD (n=2909)	MAFLD (n=3087)	P value
Basic characteristics					
Sex(male),n (%)		3663 (61.23)	1656 (57.08)	2007 (65.14)	<0.001
Age, years		58.56 ± 13.82	61.15 ± 13.46	56.25 ± 13.74	<0.001
Disease duration, years		7.09 ± 6.72	8.20 ± 7.27	6.11 ± 6.02	<0.001
BMI, kg/m²		24.56 ± 3.83	23.05 ± 3.63	25.91 ± 3.48	<0.001
SBP, mmHg		134.28 ± 21.95	134.23 ± 23.46	134.32 ± 20.56	0.132
DBP, mmHg		81.45 ± 13.05	79.59 ± 13.49	83.06 ± 12.45	<0.001
Laboratory parameters					
FBG, mmol/L		9.55 ± 4.54	9.01 ± 4.39	10.03 ± 4.62	<0.001
Fasting insulin, μU/mL		12.91 ± 45.46	12.81 ± 55.84	13.00 ± 34.81	0.522
HbA1c, %		9.38 ± 2.51	9.23 ± 2.69	9.51 ± 2.33	<0.001
TG, mmol/L		2.35 ± 3.27	1.69 ± 1.86	2.93 ± 4.05	<0.001
TC, mmol/L		4.54 ± 1.38	4.30 ± 1.21	4.75 ± 1.48	<0.001
HDL-C, mmol/L		1.04 ± 0.30	1.10 ± 0.32	0.99 ± 0.27	<0.001
LDL-C, mmol/L		2.64 ± 1.02	2.49 ± 0.97	2.77 ± 1.05	<0.001
ALP, U/L		81.25 ± 40.63	82.46 ± 51.55	80.16 ± 27.34	0.014
Ca, mmol/L		2.22 ± 0.14	2.20 ± 0.14	2.24 ± 0.14	<0.001
P, mmol/L		1.10 ± 0.20	1.08 ± 0.20	1.11 ± 0.20	<0.001
UA, μmol/L		316.83 ± 102.73	295.56 ± 100.08	335.82 ± 101.36	<0.001
TP, g/L		66.50 ± 6.50	65.34 ± 6.42	67.55 ± 6.40	<0.001
GLB, g/L		26.99 ± 4.43	26.82 ± 4.49	27.14 ± 4.37	0.703
DBIL, μmol/L		3.94 ± 4.71	4.06 ± 6.55	3.83 ± 1.92	0.05
IBIL, μmol/L		8.19 ± 4.05	7.76 ± 4.03	8.58 ± 4.04	<0.001
Scr, μmol/L		71.27 ± 53.60	74.11 ± 58.70	68.74 ± 48.48	<0.001
eGFR, mL/min/1.73 m²		98.30 ± 25.03	94.45 ± 26.49	101.70 ± 23.16	<0.001
ALT, U/L		28.98 ± 31.58	23.20 ± 25.79	34.16 ± 35.20	<0.001
AST, U/L		25.44 ± 22.39	22.99 ± 18.89	27.63 ± 24.91	<0.001
GGT, U/L		46.11 ± 86.37	39.97 ± 74.47	51.61 ± 95.48	<0.001
WBC, ×10^9^L		6.69 ± 3.46	6.67 ± 4.42	6.72 ± 2.29	0.006
NEUT, ×10^9^L		4.17 ± 2.37	4.27 ± 2.74	4.09 ± 1.97	<0.001
LYM, ×10^9^L		1.90 ± 1.80	1.79 ± 2.50	2.01 ± 0.71	<0.001
MONO, ×10^9^L		0.44 ± 0.18	0.43 ± 0.20	0.44 ± 0.16	0.055
PLT, ×10^9^L		208.89 ± 66.54	204.08 ± 68.07	213.20 ± 64.85	0.001
RBC, ×10¹²/L		4.52 ± 0.75	4.34 ± 0.74	4.67 ± 0.73	<0.001
HGB, g/L		136.08 ± 22.86	130.90 ± 22.60	140.72 ± 22.08	<0.001
hsCRP, mg/L		8.64 ± 24.21	10.39 ± 28.63	7.09 ± 19.36	<0.001
AFP, ng/mL		4.86 ± 81.96	6.22 ± 113.88	3.72 ± 38.39	0.898
CEA, ng/mL		3.37 ± 6.32	3.82 ± 9.01	2.98 ± 2.17	0.001
CA19-9, U/mL		24.35 ± 129.14	30.68 ± 189.02	19.04 ± 25.59	0.045
Insulin resistance-related indices					
TyG		9.37 ± 0.94	9.07 ± 0.84	9.64 ± 0.94	<0.001
TyG-BMI		231.03 ± 47.61	209.54 ± 40.62	250.25 ± 45.13	<0.001
METS-IR		42.04 ± 9.28	38.04 ± 8.09	45.61 ± 8.81	<0.001
CHG		5.82 ± 0.64	5.65 ± 0.62	5.97 ± 0.62	<0.001
CTI		9.69 ± 1.15	9.36 ± 1.09	9.99 ± 1.13	<0.001
HOMA-IR		4.53 ± 19.24	4.22 ± 24.43	4.81 ± 12.95	0.007
AIP		0.59 ± 0.36	0.46 ± 0.33	0.70 ± 0.36	<0.001
SHR		0.45 ± 1.22	0.42 ± 1.22	0.48 ± 1.22	0.025
Medical history					
Smoking, n (%)		2264 (37.76)	979 (33.65)	1285 (41.63)	<0.001
Drinking, n (%)		2100 (35.02)	931 (32.00)	1169 (37.87)	<0.001
Hypertension, n (%)		3240 (54.04)	1566 (53.83)	1674 (54.23)	0.779
Coronary heart disease, n (%)		817 (13.63)	439 (15.09)	378 (12.24)	0.002
Cerebral infarction, n (%)		1147 (19.13)	678 (23.31)	469 (15.19)	<0.001
Diabetic complications					
Diabetic kidney disease, n (%)		1095 (18.26)	635 (21.83)	460 (14.90)	<0.001
Diabetic retinopathy, n (%)		872 (14.54)	512 (17.60)	360 (11.66)	<0.001
Peripheral artery disease, n (%)		1959 (32.67)	985 (33.86)	974 (31.55)	0.06
Diabetic peripheral neuropathy, n (%)		3262 (54.40)	1656 (56.93)	1606 (52.02)	<0.001
Osteopenia, n (%)		822 (13.71)	410 (14.09)	412 (13.35)	0.421
Medication use					
Statin use, n (%)		1418 (23.65)	786 (27.02)	632 (20.47)	<0.001
Fibrate use, n (%)		1011 (16.86)	563 (19.35)	448 (14.51)	<0.001
Ezetimibe use, n (%)		69 (1.15)	45 (1.55)	24 (0.78)	0.008
Other antihypertensive drug use, n (%)		4002 (66.74)	1956 (67.24)	2046 (66.28)	0.446
ARB use, n (%)		289 (4.82)	53 (1.82)	236 (7.64)	<0.001
CCB use, n (%)		4177 (69.66)	1987 (68.31)	2190 (70.94)	0.028
Insulin use, n (%)		117 (1.95)	67 (2.30)	50 (1.62)	0.069
SGLT2i use, n (%)		3031 (50.55)	1447 (49.74)	1584 (51.31)	0.234
Metformin use, n (%)		2161 (36.04)	991 (34.07)	1170 (37.90)	0.002
GLP-1RA use, n (%)		1974 (32.92)	945 (32.49)	1029 (33.33)	0.502
DPP-4i use, n (%)		4448 (74.18)	2176 (74.80)	2272 (73.60)	0.301
SU use, n (%)		1253 (20.90)	476 (16.36)	777 (25.17)	<0.001
Glinide use, n (%)		2527 (42.14)	912 (31.35)	1615 (52.32)	<0.001
TZD use, n (%)		12 (0.20)	2 (0.07)	10 (0.32)	0.055
AGI use, n (%)		290 (4.84)	162 (5.57)	128 (4.15)	0.012

Values are presented as mean ± standard deviation, median interquartile range, or n (%), as appropriate.

BMI, body mass index; SBP, systolic blood pressure; DBP, diastolic blood pressure; FBG, fasting blood glucose; FINS, fasting insulin; HbA1c, glycated hemoglobin; TG, triglycerides; TC, total cholesterol; HDL-C, high-density lipoprotein cholesterol; LDL-C, low-density lipoprotein cholesterol; ALP, alkaline phosphatase; UA, uric acid; DBIL, direct bilirubin; IBIL, indirect bilirubin; Scr, serum creatinine; eGFR, estimated glomerular filtration rate; ALT, alanine aminotransferase; AST, aspartate aminotransferase; GGT, gamma-glutamyl transferase; WBC, white blood cell count; NEUT, neutrophil count; LYM, lymphocyte count; MONO, monocyte count; PLT, platelet count; RBC, red blood cell count; HGB, hemoglobin; hsCRP, high-sensitivity C-reactive protein; AFP, alpha-fetoprotein; CEA, carcinoembryonic antigen; CA19-9, carbohydrate antigen 19-9; TyG, triglyceride-glucose index; TyG-BMI, triglyceride-glucose body mass index; METS-IR, metabolic score for insulin resistance; CHG, cholesterol, high-density lipoprotein cholesterol, and glucose index; CTI, C-reactive protein–triglyceride–glucose index; HOMA-IR, homeostasis model assessment of insulin resistance; AIP, atherogenic index of plasma; SHR, stress hyperglycemia ratio; HTN, hypertension; CHD, coronary heart disease; CI, cerebral infarction; DKD, diabetic kidney disease; DR, diabetic retinopathy; PAD, peripheral artery disease; DPN, diabetic peripheral neuropathy; ARB, angiotensin receptor blocker; CCB, calcium channel blocker; SGLT2i, sodium-glucose cotransporter 2 inhibitor; GLP-1RA, glucagon-like peptide-1 receptor agonist; DPP-4i, dipeptidyl peptidase-4 inhibitor; SU, sulfonylurea; TZD, thiazolidinedione; AGI, alpha-glucosidase inhibitor.

Compared with patients without MAFLD, those with MAFLD were younger, had a shorter duration of diabetes, and had a higher BMI. The MAFLD group also exhibited a generally less favorable metabolic profile, characterized by higher FBG, HbA1c, TG, TC, and LDL-C levels and lower HDL-C levels. Significant between-group differences were also observed in several liver function, renal function, inflammatory, and hematological parameters (**Table 1**).

Lifestyle factors, comorbidities, diabetes-related complications, and medication use also differed between the two groups. In particular, smoking and alcohol consumption were more common among patients with MAFLD, whereas several diabetes-related complications, including diabetic kidney disease, diabetic retinopathy, and diabetic peripheral neuropathy, were less prevalent. Significant differences were also observed in the use of glucose-lowering, lipid-lowering, and antihypertensive medications between the two groups (**Table 1**).

### 3.2 Associations of insulin resistance-related indices with MAFLD

As shown in **Table 1**, all eight-insulin resistance-related indices differed significantly between patients with and without MAFLD (all *P* < 0.01).

To facilitate comparison across indices measured on different scales, each index was standardized, and ORs were estimated per 1-SD increase (**Table 2**). All eight indices were significantly associated with prevalent MAFLD. AIP showed the largest standardized association estimate (OR = 2.137, 95% CI: 2.007–2.275), followed by TyG (OR = 2.057, 95% CI: 1.934–2.187), CHG (OR = 1.717, 95% CI: 1.615–1.824), and CTI (OR = 1.676, 95% CI: 1.585–1.773). The associations were comparatively smaller for METS-IR (OR = 1.273, 95% CI: 1.209–1.340), TyG-BMI (OR = 1.270, 95% CI: 1.206–1.336), HOMA-IR (OR = 1.102, 95% CI: 1.020–1.190), and SHR (OR = 1.060, 95% CI: 1.007–1.115).

**Table 2:** Associations and discriminative performance of insulin resistance-related indices for prevalent MAFLD.

Variable	OR per 1-SD increase (95% CI)	P value	AUC
TyG	2.057 (1.934–2.187)	<0.001	0.682
TyG-BMI	1.270 (1.206–1.336)	<0.001	0.590
METS-IR	1.273 (1.209–1.340)	<0.001	0.578
CHG	1.717 (1.615–1.824)	<0.001	0.641
CTI	1.676 (1.585–1.773)	<0.001	0.640
HOMA-IR	1.102 (1.020–1.190)	0.013	0.678
AIP	2.137 (2.007–2.275)	<0.001	0.692
SHR	1.060 (1.007–1.115)	0.026	0.545

ORs are expressed per 1-standard deviation (SD) increase in each index to facilitate comparison of association magnitudes across indices with different measurement scales.

TyG, triglyceride-glucose index; TyG-BMI, triglyceride-glucose body mass index; METS-IR, metabolic score for insulin resistance; CHG, cholesterol, high-density lipoprotein cholesterol, and glucose index; CTI, C-reactive protein–triglyceride–glucose index; HOMA-IR, homeostasis model assessment of insulin resistance; AIP, atherogenic index of plasma; SHR, stress hyperglycemia ratio; OR, odds ratio; CI, confidence interval.

ROC analysis showed that AIP had the highest AUC among the eight indices (AUC = 0.692), followed by TyG (AUC = 0.682), HOMA-IR (AUC = 0.678), CHG (AUC = 0.641), and CTI (AUC = 0.640). Overall, the discriminative performance of the individual indices was modest, with AIP showing the highest, although moderate, discrimination for prevalent MAFLD.

### 3.3 Multivariable analysis and dose–response association of AIP with MAFLD

Given that AIP showed the largest standardized association estimate and the highest AUC among the eight indices, it was selected for further analysis. LASSO regression was applied for covariate selection (**Supplementary Figures S1A, B**; **Supplementary Table S4**). Nine variables with non-zero coefficients were retained, including diabetes duration, BMI, FBG, LDL-C, ALT, UA, eGFR, diabetic retinopathy, and glinide use. AIP was treated as the primary exposure and was not included in the LASSO selection procedure.

In the subsequent multivariable logistic regression model incorporating AIP and the nine selected covariates, AIP remained independently associated with prevalent MAFLD (OR = 4.968, 95% CI: 4.100–6.035, P < 0.001) (**Table 3**). The model showed an AUC of 0.758 (95% CI: 0.746–0.771). After internal validation using 1,000 bootstrap resamples, the optimism-corrected AUC was 0.756, indicating limited apparent optimism (**Supplementary Table S5**). Calibration, decision curve analysis, and clinical impact curve analyses are presented in **Figure 2**.

**Table 3:** Multivariable logistic regression model for prevalent MAFLD.

Variable	β	SE	OR	95% CI	P value
AIP	1.603	0.099	4.968	4.100–6.035	<0.001
Diabetes duration	−0.007	0.005	0.993	0.984–1.002	0.122
BMI	0.061	0.008	1.063	1.048–1.080	<0.001
FBG	−0.004	0.007	0.996	0.983–1.010	0.571
LDL-C	0.173	0.031	1.189	1.120–1.262	<0.001
ALT	0.004	0.001	1.004	1.002–1.006	<0.001
UA	0.0015	0.0003	1.002	1.001–1.002	<0.001
eGFR	0.013	0.001	1.014	1.011–1.016	<0.001
Diabetic retinopathy	−0.310	0.085	0.733	0.621–0.866	<0.001
Glinide use	0.643	0.060	1.902	1.693–2.138	<0.001

Multivariable logistic regression was performed with prevalent MAFLD as the outcome. AIP was included as the primary exposure, and the remaining covariates were selected using LASSO regression. ORs for continuous variables represent the change in odds of prevalent MAFLD per one-unit increase in the corresponding variable.

MAFLD, metabolic dysfunction-associated fatty liver disease; AIP, atherogenic index of plasma; β regression coefficient; SE, standard error; OR, odds ratio; CI, confidence interval; BMI, body mass index; FBG, fasting blood glucose; LDL-C, low-density lipoprotein cholesterol; ALT, alanine aminotransferase; UA, uric acid; eGFR, estimated glomerular filtration rate.

**Figure 2:**
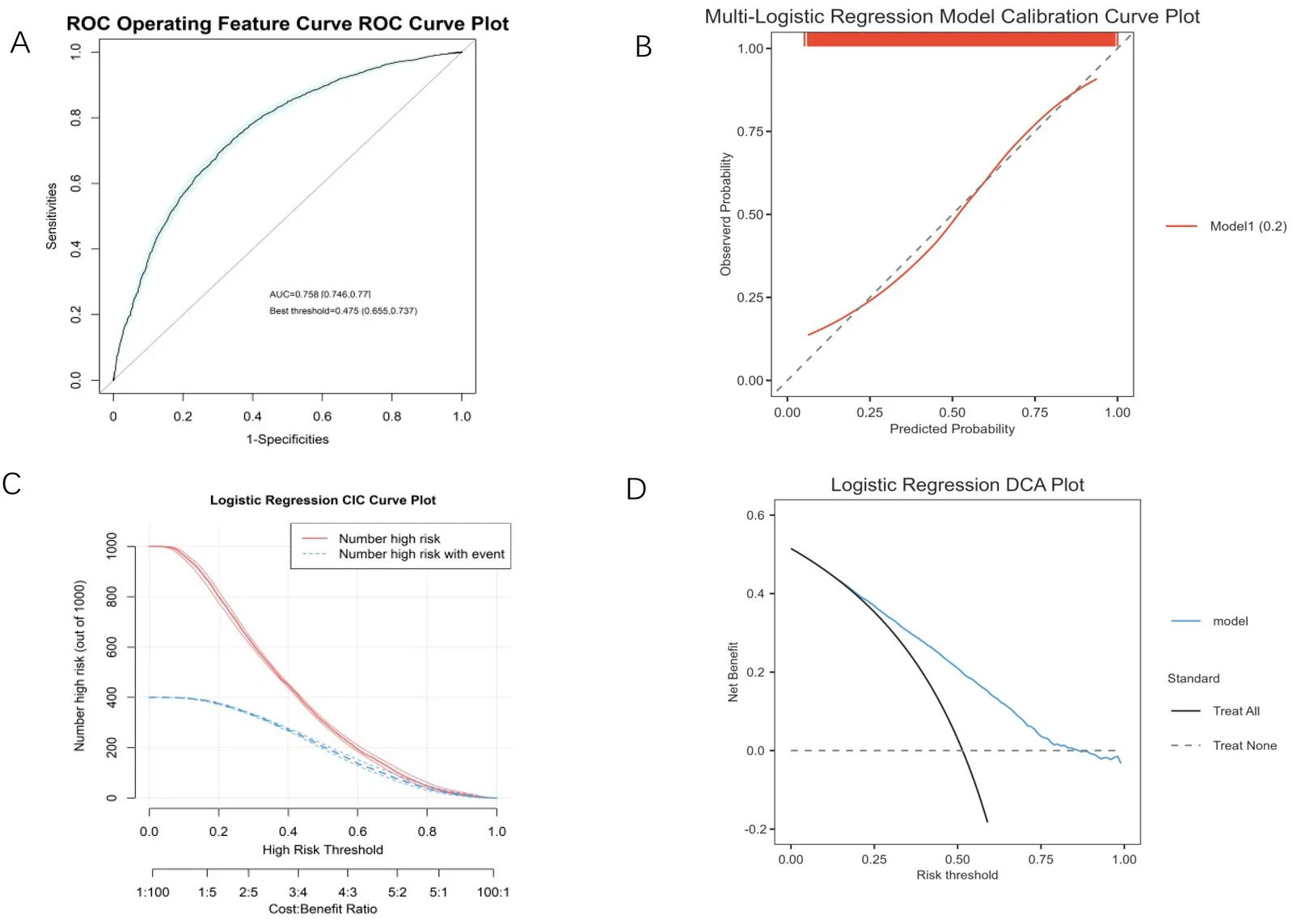
Performance evaluation of the multivariable model for prevalent MAFLD. **(A)** ROC curve showing an AUC of 0.758 (95% CI: 0.746–0.771); the optimism-corrected AUC after 1,000 bootstrap resamples was 0.756. **(B)** Calibration curve comparing predicted and observed probabilities. **(C)** Clinical impact curve showing the number of individuals classified as high risk and the number of true-positive cases across threshold probabilities. **(D)** Decision curve analysis showing the net benefit of the model compared with treat-all and treat-none strategies. The model incorporated AIP, diabetes duration, BMI, FBG, LDL-C, ALT, UA, eGFR, diabetic retinopathy, and glinide use.

The RCS analyses of TyG, CHG, CTI, and AIP are presented in **Supplementary Figure S2**. Given that AIP showed the largest standardized association estimate and the highest discriminative performance among the evaluated indices, we further examined its association with prevalent MAFLD using an adjusted RCS model. Adjusted RCS analysis further demonstrated a significant association between AIP and prevalent MAFLD (P for overall < 0.001), with evidence of nonlinearity (P for nonlinearity = 0.040) (**Figure 3**). The spline model was adjusted for diabetes duration, BMI, FBG, LDL-C, ALT, UA, eGFR, diabetic retinopathy, and glinide use. The association generally increased with increasing AIP levels, although the shape of the association was nonlinear.

**Figure 3:**
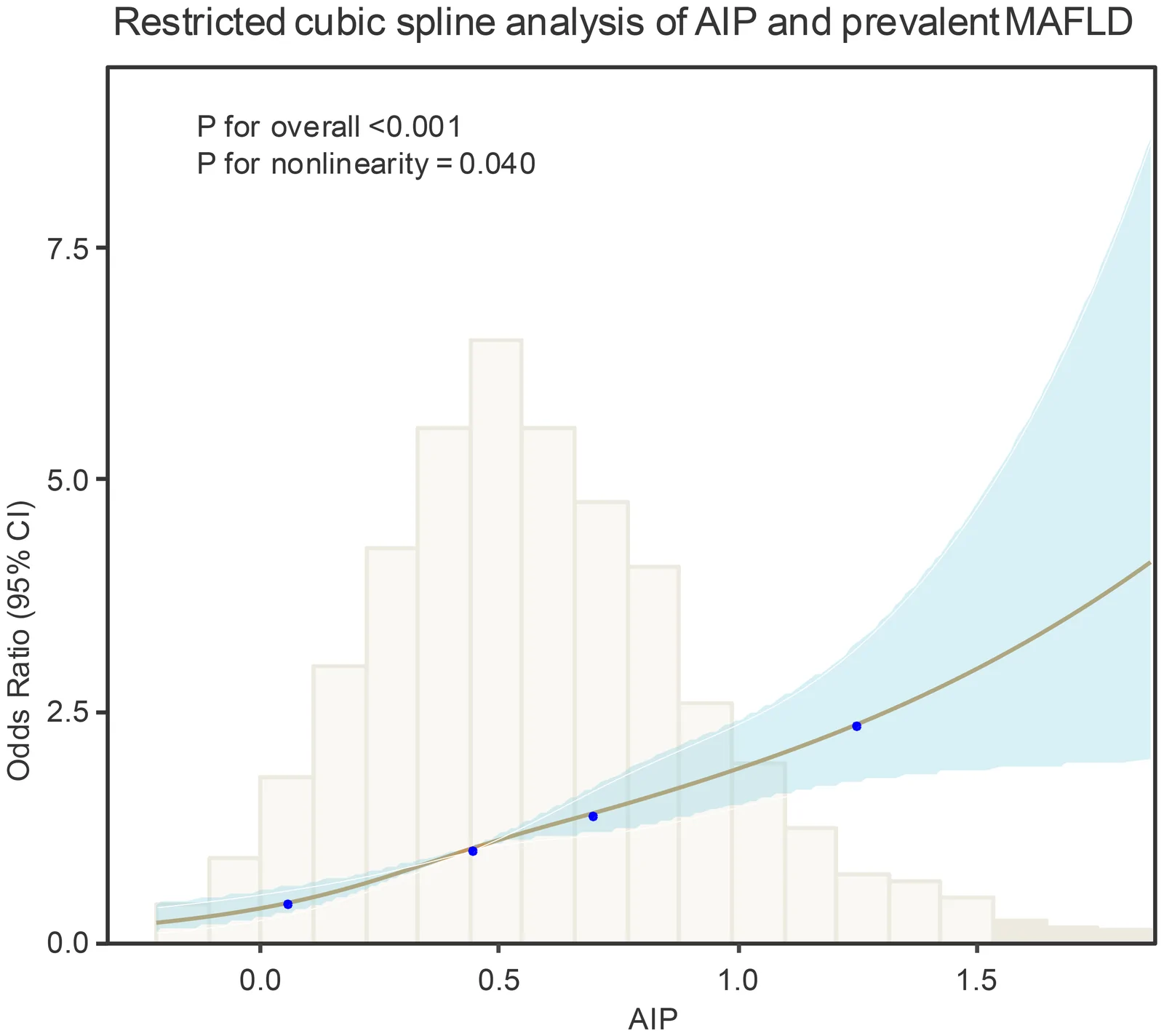
Restricted cubic spline analysis of the association between AIP and prevalent MAFLD. Restricted cubic spline logistic regression was fitted using four knots located at AIP values of 0.0505, 0.4360, 0.6884, and 1.2466, with the second knot (AIP = 0.4360) serving as the reference (OR = 1.00). The model was adjusted for diabetes duration, BMI, FBG, LDL-C, ALT, UA, eGFR, diabetic retinopathy, and glinide use. The solid line and shaded area represent the estimated OR and 95% CI, respectively. The histogram shows the distribution of AIP. The overall association was statistically significant (P for overall < 0.001), with evidence of nonlinearity (P for nonlinearity = 0.040).

### 3.4 Modification of the association between AIP and MAFLD by diabetes duration

To explore whether the association between AIP and prevalent MAFLD varied according to diabetes duration, stratified and interaction analyses were performed. In the primary stratified analysis, diabetes duration was categorized as <5 and ≥5 years. Sensitivity analyses using the original three categories (<5, 5–10, and ≥10 years) were additionally performed.

In the stratified ROC analysis, AIP showed an AUC of 0.678 among patients with diabetes duration <5 years and 0.694 among those with duration ≥5 years (**Figure 4**). The between-group difference in the AUC of AIP was not statistically significant (P = 0.236). Within the <5-year group, the discriminative performance of AIP was comparable to that of TyG (P = 0.158) and CHG (P = 0.146), but differed significantly from that of CTI (P < 0.001). In the ≥5-year group, AIP showed significantly higher discriminative performance than TyG, CHG, and CTI (all P < 0.001) (**Table 4**).

**Figure 4:**
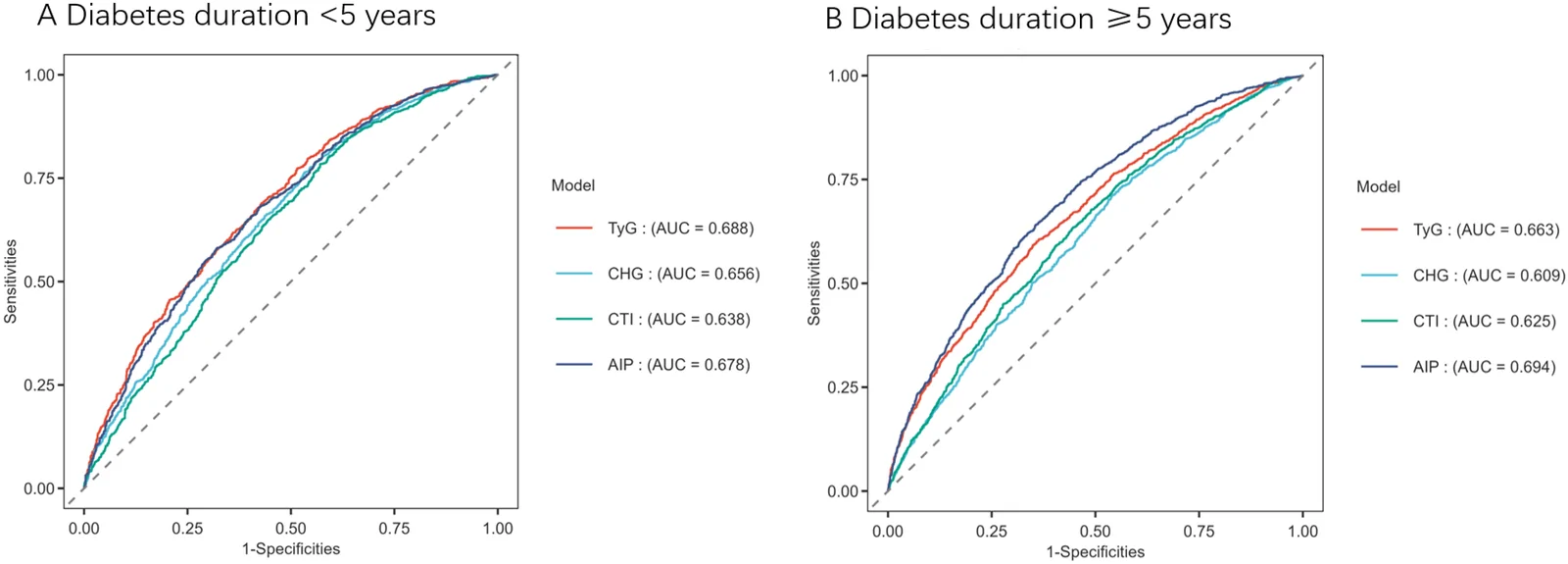
Receiver operating characteristic (ROC) curves comparing the discriminative performance of TyG, CHG, CTI, and AIP for identifying MAFLD in patients with diabetes duration <5 years **(A)** and ≥5 years **(B)**. The area under the ROC curve (AUC) for each index is shown in the legend.

**Table 4:** Comparison of the diagnostic performance of AIP and other insulin resistance indices according to diabetes duration.

Comparison	AUC (<5 years)	*P*	AUC (≥5 years)	*P*
AIP vs. TyG	0.678 vs. 0.688	0.158	0.694 vs. 0.663	<0.001
AIP vs. CHG	0.678 vs. 0.656	0.146	0.694 vs. 0.609	<0.001
AIP vs. CTI	0.678 vs. 0.638	<0.001	0.694 vs. 0.625	<0.001

AIP, atherogenic index of plasma; TyG, triglyceride-glucose index; CHG, cholesterol, high-density lipoprotein cholesterol, and glucose index; CTI, C-reactive protein–triglyceride–glucose index; AUC, area under the curve.

Sensitivity analyses using the original three duration categories showed that AIP remained significantly associated with prevalent MAFLD across all three groups. The standardized ORs per 1-SD increase in AIP were 1.970 (95% CI: 1.798–2.158) for <5 years, 2.171 (95% CI: 1.880–2.508) for 5–10 years, and 2.215 (95% CI: 1.985–2.472) for ≥10 years (**Supplementary Table S6**). The corresponding AUCs were 0.678, 0.697, and 0.689, respectively.

Interaction analyses further showed significant interactions between AIP and diabetes duration when duration was modeled continuously (P for interaction = 0.031) and categorically (<5 vs. ≥5 years; P for interaction = 0.001) (**Table 5**), suggesting that the association between AIP and prevalent MAFLD may vary according to diabetes duration.

**Table 5:** Interaction analysis between AIP and diabetes duration for the risk of MAFLD.

Interaction term	OR (95 CI)	P
AIP × Diabetes duration (continuous)	1.035 (1.003–1.0671)	0.031
AIP × Duration group (≥5 vs <5 years)	1.691 (1.135–2.516)	0.001

AIP, atherogenic index of plasma; CI, confidence interval; OR, odds ratio.

Adjusted for all covariates selected by LASSO regression (see **Supplementary Table S1**). Interaction terms were created by multiplying AIP with diabetes duration (continuous) or diabetes duration group (<5 vs. ≥5 years). ORs are presented with 95% confidence intervals.

## 4 Discussion

In this large cross-sectional study of 5,996 patients with T2DM, we comprehensively compared the associations and discriminative performance of multiple IR-related indices for prevalent MAFLD. Four principal findings emerged. First, all eight IR-related indices were significantly associated with prevalent MAFLD. Second, after standardization to account for differences in measurement scales, AIP showed the largest association estimate per 1-SD increase and the highest discriminative performance among the evaluated indices, although its discriminative ability was moderate. Third, adjusted restricted cubic spline analysis demonstrated a significant nonlinear association between AIP and prevalent MAFLD. Finally, AIP remained independently associated with prevalent MAFLD after multivariable adjustment, and significant interaction analyses indicated that the association between AIP and MAFLD varied according to diabetes duration.

Insulin resistance is considered the fundamental pathophysiological link between T2DM and MAFLD. Impaired insulin signaling disrupts glucose and lipid homeostasis, promoting excessive adipose tissue lipolysis, increased hepatic free fatty acid influx, enhanced *de novo* lipogenesis, and reduced fatty acid oxidation, ultimately leading to hepatic steatosis (22). Moreover, adipose tissue dysfunction results in the release of adipokines, inflammatory cytokines, chemokines, and lipotoxic metabolites, which further aggravate systemic and hepatic insulin resistance. Given the pivotal role of IR in the development of MAFLD, several easily accessible surrogate indices have been proposed as practical alternatives to direct assessment of insulin sensitivity for the early identification of individuals at high risk for MAFLD. In our study, all eight IR-related indices were significantly associated with prevalent MAFLD. After standardization, AIP showed the largest association estimate per 1-SD increase and the highest AUC among the evaluated indices. In stratified analyses, AIP showed significantly higher discrimination than TyG, CHG, and CTI in patients with diabetes duration ≥5 years, whereas its performance was comparable to TyG and CHG in those with diabetes duration <5 years. These findings suggest that AIP may provide useful information for identifying prevalent MAFLD in patients with T2DM.

The superior performance of AIP is biologically plausible (11, 23, 24). Unlike traditional insulin resistance markers, AIP integrates triglyceride and HDL-cholesterol concentrations, thereby simultaneously reflecting lipid metabolism disorders, insulin resistance, and the presence of atherogenic dyslipidemia. Since lipid accumulation is a hallmark of MAFLD pathogenesis, AIP may more directly reflect hepatic metabolic dysfunction than glucose-based indices alone. Furthermore, our restricted cubic spline analysis demonstrated a nonlinear increase in MAFLD risk with increasing AIP, indicating that the association became progressively stronger at higher AIP levels. Interestingly, baseline analyses showed that patients with MAFLD were less likely to receive lipid-lowering therapy, including statins, ezetimibe, and fibrates, despite exhibiting a more adverse lipid profile. Although causality cannot be inferred from this cross-sectional study, this observation may partly explain the persistently elevated AIP observed in the MAFLD group.

Several mechanisms may explain this observation. Prolonged diabetes duration is accompanied by sustained hyperglycemia, progressive insulin resistance, and cumulative lipid metabolic disturbances (25). Chronic insulin resistance promotes persistent adipose tissue lipolysis and increases the flux of free fatty acids into the liver, resulting in excessive hepatic triglyceride accumulation and worsening steatosis. In parallel, long-standing diabetes is associated with progressive β-cell dysfunction (26), chronic low-grade inflammation, oxidative stress (27), and mitochondrial dysfunction (28), all of which further impair hepatic lipid metabolism and accelerate MAFLD progression (29). Because AIP reflects the combined effects of hypertriglyceridemia and reduced HDL-C, it may better capture these cumulative metabolic abnormalities than glucose-based insulin resistance indices alone. Consequently, the association between AIP and MAFLD became progressively stronger as diabetes duration increased.

Overall, our findings demonstrate that AIP is the insulin resistance-related index most strongly associated with MAFLD in patients with T2DM. Moreover, diabetes duration modifies this association, with stronger predictive value observed in patients with longer disease duration. Given its simplicity, low cost, and widespread availability in routine clinical practice, AIP may represent a valuable tool for early identification and risk stratification of MAFLD among individuals with T2DM.

Several limitations should be acknowledged. First, the cross-sectional design precludes causal inference between AIP and MAFLD. Second, this was a single-center study of hospitalized patients with T2DM, which may limit the generalizability of our findings to broader T2DM populations. Third, the exclusion of patients with incomplete essential clinical, laboratory, or imaging data may have introduced selection bias, although differences between included and excluded participants were further assessed. Fourth, MAFLD was diagnosed based on clinical and imaging criteria rather than histological confirmation, and residual confounding cannot be completely excluded. Finally, although bootstrap internal validation showed limited optimism in the multivariable model, external validation in independent populations is still required to confirm its robustness and generalizability.

## 5 Conclusion

Among multiple insulin resistance-related indices, AIP showed the largest standardized association estimate and the highest discriminative performance for prevalent MAFLD in patients with T2DM. The association between AIP and MAFLD was nonlinear and varied according to diabetes duration. These findings support AIP as a simple and clinically accessible metabolic marker associated with prevalent MAFLD in patients with T2DM.

## Data Availability

The raw data supporting the conclusions of this article will be made available by the authors, without undue reservation.

## Glossary

AGIs: α-Glucosidase inhibitors
AIP: Atherogenic index of plasma
ALT: Alanine aminotransferase
ARBs: Angiotensin II receptor blockers
AST: Aspartate aminotransferase
AUC: Area under the receiver operating characteristic curve
BMI: Body mass index
Ca: Serum calcium
CAD: Coronary artery disease
CCBs: Calcium channel blockers
CEA: Carcinoembryonic antigen
CHG: Cholesterol, high-density lipoprotein cholesterol, and glucose index
CI: Confidence interval
CTI: C-reactive protein–triglyceride–glucose index
DBP: Diastolic blood pressure
DCA: Decision curve analysis
DKD: Diabetic kidney disease
DPN: Diabetic peripheral neuropathy
DR: Diabetic retinopathy
eGFR: Estimated glomerular filtration rate
FBG: Fasting blood glucose
FINS: Fasting insulin
GGT: Gamma-glutamyl transferase
HbA1c: Glycated hemoglobin
HGB: Hemoglobin
HDL-C: High-density lipoprotein cholesterol
HOMA-IR: Homeostasis model assessment of insulin resistance
hs-CRP: High-sensitivity C-reactive protein
IBIL: Indirect bilirubin
IQR: Interquartile range
IR: Insulin resistance
LASSO: Least absolute shrinkage and selection operator
LDL-C: Low-density lipoprotein cholesterol
LYM: Lymphocyte count
MAFLD: Metabolic dysfunction-associated fatty liver disease
METS-IR: Metabolic score for insulin resistance
NEUT: Neutrophil count
OR: Odds ratio
P: Phosphorus
PLT: Platelet count
RBC: Red blood cell count
RCS: Restricted cubic spline
ROC: Receiver operating characteristic
SBP: Systolic blood pressure
Scr: Serum creatinine
SD: Standard deviation
SHR: Stress hyperglycemia ratio
SUs: Sulfonylureas
T2DM: Type 2 diabetes mellitus
TC: Total cholesterol
TG: Triglycerides
TP: Total protein
TyG: Triglyceride-glucose index
TyG-BMI: Triglyceride-glucose body mass index
UA: Uric acid
WBC: White blood cell count
