# Supplementary material for: Diabetes duration modifies the association between AIP and MAFLD in type 2 diabetes
Source: Front Endocrinol (Lausanne). 2026 Oct 6;17:1952511. doi: 10.3389/fendo.2026.1952511 (PMC13680141; doi:10.3389/fendo.2026.1952511)
Supplement: Supplementary file 1 [file DataSheet1.pdf]

Supplementary Table S1. Components and metabolic characteristics of insulin resistance-related surrogate indices

Index	Main components	Insulin required	Main metabolic characteristics
**TyG**	TG, FBG	No	Integrates fasting triglyceride and glucose levels as a surrogate measure of insulin resistance
**TyG-BMI**	TG, FBG, BMI	No	Extends TyG by incorporating adiposity, thereby combining metabolic disturbance with overall obesity
**METS-IR**	FBG, TG, HDL-C, BMI	No	Integrates glycemic status, lipid profile, and adiposity to reflect insulin resistance
**CHG**	TC, FBG, HDL-C	No	Combines glycemic status with the balance between total and HDL cholesterol
**CTI**	TG, FBG, hs-CRP	No	Integrates glucose–lipid metabolism with systemic inflammation
**HOMA-IR**	FBG, fasting insulin	**Yes**	Estimates insulin resistance from fasting glucose and insulin concentrations
**SHR**	FBG, HbA1c	No	Reflects acute glycemia relative to estimated chronic glycemic exposure
**AIP**	TG, HDL-C	No	Reflects the balance between atherogenic and protective lipid fractions and is associated with an atherogenic lipid profile

Supplementary Table S2. Candidate variables entered into the LASSO regression and variables retained in the final model

Category	Candidate variables entered into LASSO
**Demographic and lifestyle factors**	Sex, age, smoking, drinking
**Diabetes-related characteristics**	Diabetes duration
**Anthropometric and blood pressure parameters**	BMI, SBP, DBP
**Glycemic parameters**	FBG, HbA1c
**Lipid parameters**	LDL-C
**Liver function parameters**	ALT, AST, GGT, ALP
**Renal/metabolic parameters**	UA, eGFR
**Hematological parameters**	WBC, PLT
**Cardiovascular and diabetes-related comorbidities/complications**	CHD, CI, DKD, DR
**Glucose-lowering medications**	Metformin, SU, glinide, SGLT2i, GLP-1RA, DPP-4i, AGI, insulin
**Lipid-lowering medications**	Statin, fibrate, ezetimibe
**Antihypertensive medications**	Antihypertensive medication

Abbreviations: AGI, α-glucosidase inhibitor; ALT, alanine aminotransferase; AST, aspartate

aminotransferase; BMI, body mass index; CHD, coronary heart disease; CI, cerebral infarction; DBP, diastolic blood pressure; DKD, diabetic kidney disease; DPP-4i, dipeptidyl peptidase-4 inhibitor; DR, diabetic retinopathy; eGFR, estimated glomerular filtration rate; FBG, fasting blood glucose; GGT, γ-glutamyl transferase; GLP-1RA, glucagon-like peptide-1 receptor agonist; HbA1c, glycated hemoglobin; LDL-C, low-density lipoprotein cholesterol; PLT, platelet count; SBP, systolic blood pressure; SGLT2i, sodium-glucose cotransporter-2 inhibitor; SU, sulfonylurea; UA, uric acid; WBC, white blood cell count.

Supplementary Table S3. Missingness and baseline characteristics of included and excluded participants

Characteristics	Missing %	Included participants (n=5,996)	Excluded participants (n=3,213)	P value	SMD
**Basic characteristics**					
Sex (male), n (%)	0.2	3,663 (61.23)	2,058 (64.09)	0.008	0.059
Age, years	0.0	59.70 ± 14.36	59.33 ± 14.26	0.238	0.026
Disease duration, years	0.0	6.97 ± 6.80	8.05 ± 7.28	<0.001	0.154
BMI, kg/m²	34.1	24.56 ± 3.83	24.81 ± 3.72	0.011	0.067
SBP, mmHg	15.8	136.26 ± 23.81	137.33 ± 23.69	0.229	0.045
DBP, mmHg	15.9	81.09 ± 13.91	80.31 ± 14.47	0.138	0.055
**Laboratory parameters**					
FBG, mmol/L	33.8	9.41 ± 4.59	9.88 ± 5.40	0.306	0.094
Fasting insulin, μU/mL	28.0	12.17 ± 42.28	12.22 ± 39.94	0.964	0.001
HbA1c, %	21.5	9.34 ± 2.53	9.34 ± 2.46	0.947	0.002
TG, mmol/L	33.0	2.25 ± 3.17	2.29 ± 3.08	0.873	0.012
TC, mmol/L	15.5	4.46 ± 1.37	4.31 ± 1.41	<0.001	0.106
HDL-C, mmol/L	15.3	1.05 ± 0.31	1.04 ± 0.29	0.192	0.035
LDL-C, mmol/L	15.3	2.60 ± 1.03	2.37 ± 0.93	<0.001	0.232
ALP, U/L	14.5	84.64 ± 39.84	87.58 ± 40.82	0.005	0.073
Ca, mmol/L	19.1	2.20 ± 0.14	2.16 ± 0.13	<0.001	0.361
P, mmol/L	19.7	1.09 ± 0.23	1.11 ± 0.24	0.012	0.071
UA, μmol/L	16.6	321.37 ± 109.41	314.54 ± 103.89	0.019	0.064
TP, g/L	14.1	66.35 ± 6.78	67.34 ± 6.83	<0.001	0.146
GLB, g/L	14.1	27.16 ± 4.47	27.54 ± 4.47	0.001	0.087
DBIL, μmol/L	30.6	3.86 ± 4.79	3.83 ± 2.26	0.890	0.009
IBIL, μmol/L	16.5	8.19 ± 4.10	7.94 ± 4.19	0.031	0.059
Scr, μmol/L	13.7	80.59 ± 89.13	77.64 ± 73.60	0.185	0.036
eGFR, mL/min/1.73 m²	24.6	95.19 ± 27.16	94.81 ± 26.82	0.609	0.014
ALT, U/L	14.2	29.14 ± 36.36	31.52 ± 44.35	0.018	0.059

Characteristics	Missing %	Included participants (n=5,996)	Excluded participants (n=3,213)	P value	SMD
AST, U/L	13.9	25.20 ± 25.02	26.48 ± 36.22	0.083	0.041
GGT, U/L	14.1	45.22 ± 81.47	47.30 ± 89.21	0.341	0.024
WBC, $\times 10^9$/L	0.0	6.95 ± 3.44	4.70 ± 4.15	<0.001	0.591
NEUT, $\times 10^9$/L	0.0	4.48 ± 2.82	3.00 ± 3.13	<0.001	0.498
LYM, $\times 10^9$/L	0.0	1.83 ± 1.42	1.23 ± 1.11	<0.001	0.469
MONO, $\times 10^9$/L	0.0	0.47 ± 0.22	0.34 ± 0.31	<0.001	0.465
PLT, $\times 10^9$/L	0.0	210.56 ± 69.36	138.28 ± 111.03	<0.001	0.781
RBC, $\times 10^{12}$/L	0.0	4.49 ± 0.79	3.05 ± 2.21	<0.001	0.872
HGB, g/L	0.0	135.19 ± 23.75	91.42 ± 66.59	<0.001	0.875
hsCRP, mg/L	16.5	10.13 ± 27.29	9.04 ± 23.62	0.114	0.043
AFP, ng/mL	25.9	4.32 ± 69.88	2.61 ± 3.89	0.326	0.035
CEA, ng/mL	22.7	3.45 ± 6.88	3.14 ± 2.22	0.069	0.060
CA19-9, U/mL	26.5	23.69 ± 110.93	22.64 ± 143.38	0.757	0.008
**Medical history**					
Smoking, n (%)	0.0	2,264 (37.76)	1,292 (40.21)	0.022	0.050
Drinking, n (%)	0.0	2,100 (35.02)	1,263 (39.31)	<0.001	0.089
Hypertension, n (%)	0.0	3,240 (54.04)	1,821 (56.68)	0.016	0.053
Coronary heart disease, n (%)	0.0	817 (13.63)	672 (20.92)	<0.001	0.194
Cerebral infarction, n (%)	0.0	1,147 (19.13)	507 (15.78)	<0.001	0.088
**Diabetic complications**					
Diabetic kidney disease, n (%)	0.0	1,095 (18.26)	653 (20.32)	0.017	0.052
Diabetic retinopathy, n (%)	0.0	872 (14.54)	472 (14.69)	0.873	0.004
Peripheral artery disease, n (%)	0.0	1,959 (32.67)	1,726 (53.72)	<0.001	0.435
Diabetic peripheral neuropathy, n (%)	0.0	3,262 (54.40)	2,124 (66.11)	<0.001	0.241
Osteopenia, n (%)	0.0	822 (13.71)	630 (19.61)	<0.001	0.159
**Medication use**	0.0				
Statin use, n (%)	0.0	1418 (23.65)	514 (16.00)	<0.001	0.193
Fibrate use, n (%)	0.0	1011 (16.86)	413 (12.85)	<0.001	0.113
Ezetimibe use, n (%)	0.0	69 (1.15)	13 (0.40)	<0.001	0.102
Other antihypertensive drug use, n (%)	0.0	4002 (66.74)	1368 (42.58)	<0.001	0.085
ARB use, n (%)	0.0	289 (4.82)	94 (2.93)	<0.001	0.500
CCB use, n (%)	0.0	4177 (69.66)	1413 (43.98)	<0.001	0.098
Insulin use, n (%)	0.0	117 (1.95)	134 (4.17)	<0.001	0.537

Characteristics	Missing %	Included participants (n=5,996)	Excluded participants (n=3,213)	P value	SMD
SGLT2i use, n (%)	0.0	3031 (50.55)	979 (30.47)	<0.001	0.129
Metformin use, n (%)	0.0	2161 (36.04)	722 (22.47)	<0.001	0.418
GLP-1RA use, n (%)	0.0	1974 (32.92)	636 (19.79)	<0.001	0.302
DPP-4i use, n (%)	0.0	4448 (74.18)	1419 (44.16)	<0.001	0.301
SU use, n (%)	0.0	1253 (20.90)	711 (22.13)	<0.001	0.641
Glinide use, n (%)	0.0	2527 (42.14)	1000 (31.12)	0.177	0.030
TZD use, n (%)	0.0	12 (0.20)	8 (0.25)	<0.001	0.230
AGI use, n (%)	0.0	290 (4.84)	318 (9.90)	0.806	0.010

Note: Values are presented as mean ± standard deviation, median interquartile range, or n (%), as appropriate. SMD, standardized mean difference. Variable-level missingness was assessed before exclusion of participants with incomplete essential data. SMDs were used to quantify differences in available baseline characteristics between included and excluded participants.

Abbreviations: BMI, body mass index; SBP, systolic blood pressure; DBP, diastolic blood pressure; FBG, fasting blood glucose; FINS, fasting insulin; HbA1c, glycated hemoglobin; TG, triglycerides; TC, total cholesterol; HDL-C, high-density lipoprotein cholesterol; LDL-C, low-density lipoprotein cholesterol; ALP, alkaline phosphatase; UA, uric acid; DBIL, direct bilirubin; IBIL, indirect bilirubin; Scr, serum creatinine; eGFR, estimated glomerular filtration rate; ALT, alanine aminotransferase; AST, aspartate aminotransferase; GGT, gamma-glutamyl transferase; WBC, white blood cell count; NEUT, neutrophil count; LYM, lymphocyte count; MONO, monocyte count; PLT, platelet count; RBC, red blood cell count; HGB, hemoglobin; hsCRP, high-sensitivity C-reactive protein; AFP, alpha-fetoprotein; CEA, carcinoembryonic antigen; CA19-9, carbohydrate antigen 19-9; HTN, hypertension; CHD, coronary heart disease; CI, cerebral infarction; DKD, diabetic kidney disease; DR, diabetic retinopathy; PAD, peripheral artery disease; DPN, diabetic peripheral neuropathy; ARB, angiotensin receptor blocker; CCB, calcium channel blocker; SGLT2i, sodium-glucose cotransporter 2 inhibitor; GLP-1RA, glucagon-like peptide-1 receptor agonist; DPP-4i, dipeptidyl peptidase-4 inhibitor; SU, sulfonylurea; TZD, thiazolidinedione; AGI, alpha-glucosidase inhibitor.

Supplementary Table S4. Candidate variables entered into the LASSO regression and variables retained at λ.1se

Variable	LASSO coefficient at λ.1se	Retained
Sex	0	No
Age	0	No
Diabetes duration	**−0.0155**	**Yes**
Smoking	0	No
Drinking	0	No
BMI	**0.2139**	**Yes**
SBP	0	No

Variable	LASSO coefficient at λ.1se	Retained
DBP	0	No
FBG	**0.0035**	**Yes**
HbA1c	0	No
LDL-C	**0.0352**	**Yes**
ALT	**0.0038**	**Yes**
AST	0	No
GGT	0	No
ALP	0	No
UA	**0.0003**	**Yes**
eGFR	**0.0009**	**Yes**
WBC	0	No
PLT	0	No
CHD	0	No
Cerebral infarction	0	No
DKD	0	No
Diabetic retinopathy	**−0.2345**	**Yes**
Statin use	0	No
Fibrate use	0	No
Ezetimibe use	0	No
Antihypertensive medication	0	No
Metformin use	0	No
Sulfonylurea use	0	No
**Glinide use**	**0.3348**	**Yes**
SGLT2i use	0	No
GLP-1RA use	0	No
DPP-4i use	0	No
AGI use	0	No
Insulin use	0	No

Abbreviations: AGI, α-glucosidase inhibitor; ALP, alkaline phosphatase; ALT, alanine aminotransferase; BMI, body mass index; CHD, coronary heart disease; DBP, diastolic blood pressure; DKD, diabetic kidney disease; DPP-4i, dipeptidyl peptidase-4 inhibitor; eGFR, estimated glomerular filtration rate; FBG, fasting blood glucose; GLP-1RA, glucagon-like peptide-1 receptor agonist; GGT, γ-glutamyl transferase; HbA1c, glycated hemoglobin; LDL-C, low-density lipoprotein cholesterol; PLT, platelet count; SBP, systolic blood pressure; SGLT2i, sodium-glucose cotransporter-2 inhibitor; UA, uric acid; WBC, white blood cell count.

Note: LASSO regression was applied to 35 candidate covariates. The penalty parameter was selected using the one-standard-error criterion (λ.1se). Nine variables with non-zero coefficients at λ.1se were retained for subsequent multivariable analysis: diabetes duration, BMI, FBG, LDL-C,

ALT, UA, eGFR, diabetic retinopathy, and glinide use. AIP was treated as the primary exposure of interest and was therefore not subjected to LASSO variable selection.

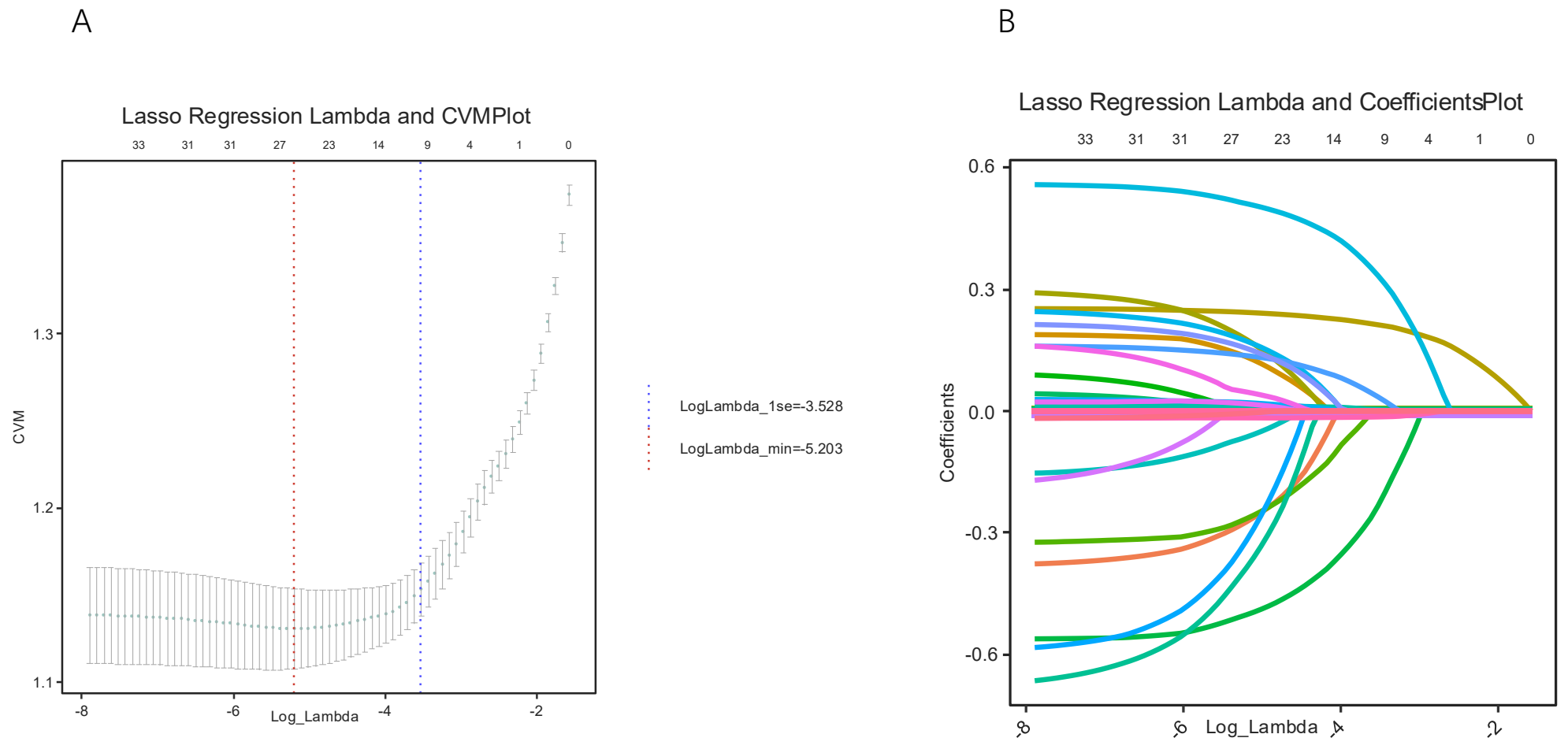

Supplementary Figure S1. Variable selection using LASSO logistic regression.
(A) Ten-fold cross-validation for selection of the optimal tuning parameter (λ) in the LASSO logistic regression. The curve represents the cross-validated mean (CVM) with standard errors across different values of log(λ). The numbers along the top indicate the number of variables with nonzero coefficients. The red dotted line represents λ.min (log λ = −5.203), which corresponds to the minimum cross-validation error, whereas the blue dotted line represents λ.1se (log λ = −3.528), the largest λ within one standard error of the minimum. **The λ.1se criterion was selected to obtain a more parsimonious model while maintaining cross-validated performance.**
(B) LASSO coefficient profiles of the candidate variables. Each colored line represents the coefficient trajectory of an individual variable as a function of log(λ). With increasing penalization, the coefficients are progressively shrunk toward zero. **Variables with nonzero coefficients at λ.1se were retained for subsequent multivariable logistic regression analysis.**

Supplementary Table S5. Bootstrap internal validation of the final multivariable model

**Performance measure**	**Estimate**
Apparent AUC	0.758
Number of bootstrap resamples	1,000
Mean optimism	0.0019
Optimism-corrected AUC	0.756
Successful bootstrap iterations	1,000/1,000

Supplementary Table S6. Association and discriminative performance of AIP for prevalent MAFLD according to the original three categories of diabetes duration

Diabetes duration	AIP OR per 1-SD (95% CI)	P value	AUC (95% CI)
<5 years	1.970 (1.798–2.158)	<0.001	0.678 (0.657–0.698)
5–10 years	2.171 (1.880–2.508)	<0.001	0.697 (0.667–0.727)
≥10 years	2.215 (1.985–2.472)	<0.001	0.689 (0.667–0.711)

Note: ORs are expressed per 1-SD increase in AIP. Diabetes duration was categorized as <5, 5–10, and ≥10 years in the exploratory analysis. AUC, area under the receiver operating characteristic curve; CI, confidence interval; OR, odds ratio; SD, standard deviation.

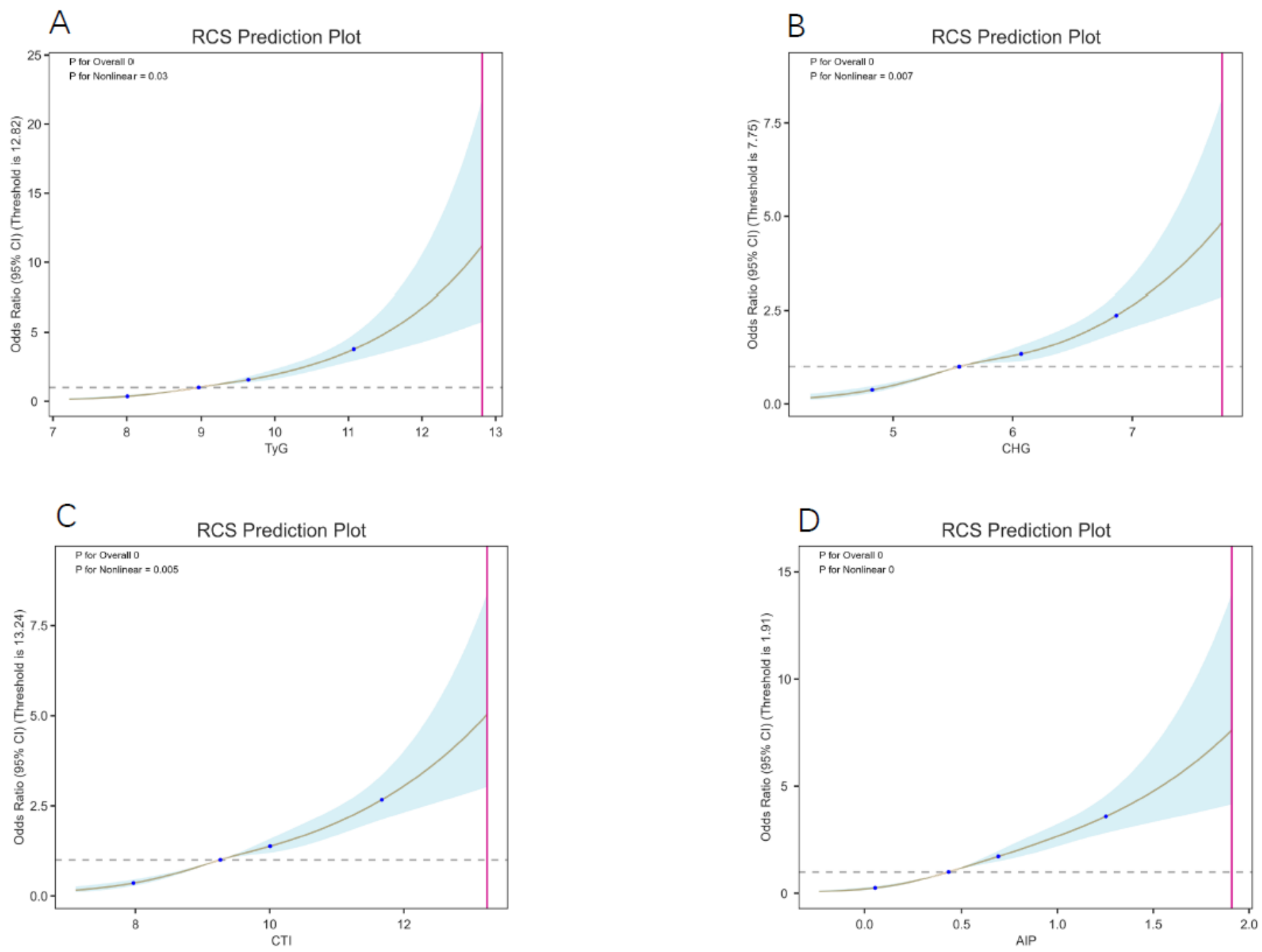

Supplementary Figure S2. Restricted cubic spline (RCS) analyses showing the associations between insulin resistance indices and the risk of MAFLD. Panels represent **(A)** TyG, **(B)** CHG, **(C)** CTI, and **(D)** AIP. Solid lines indicate the estimated odds ratios, and shaded areas represent the 95% confidence intervals. The horizontal dashed line indicates an odds ratio of 1.0, and the vertical magenta line represents the estimated threshold value identified by the RCS model.
